# Prognostic Factors Associated With Overall Survival in Breast Cancer Patients With Metastatic Spinal Disease

**DOI:** 10.7759/cureus.48909

**Published:** 2023-11-16

**Authors:** Gervith Reyes Soto, Bernardo Cacho-Díaza, Carlos Bravo-Reynab, José Raul Guerra-Mora, Carlos Ovalles, Carlos Catillo-Rangel, Manuel de Jesus Encarnacion Ramirez, Nicola Montemurro

**Affiliations:** 1 Neurosurgical Oncology, Mexico National Cancer Institute, Mexico City, MEX; 2 Functional Neurosciences, Mexico National Cancer Institute, Mexico City, MEX; 3 Experimental Surgery, National Institute of Medical Sciences and Nutrition Salvador Zubirán (INCMNSZ), Mexico City, MEX; 4 Neurosurgery, General Hospital, Durango, Durango, MEX; 5 Neurosurgery, Hospital Regional 1ro de Octubre (ISSSTE or Instituto de Seguridad y Servicios Sociales de los Trabajadores del Estado), Mexico City, MEX; 6 Neurosurgery, Peoples' Friendship University of Russia, Moscow, RUS; 7 Neurosurgery, Azienda Ospedaliero Universitaria Pisana (AOUP), Pisa, ITA

**Keywords:** surgical treatment, scoring systems, prognostic factors, spinal metastases, breast cancer

## Abstract

Introduction

The spine is the third most frequent site of metastasis, after the lungs and liver, in breast cancer patients. The current treatment modality is based on the prognosis calculated according to multiple clinical features; therefore, multiple scores have been developed to make the therapeutic decision; however, there are no specific scores to take an adequate therapeutic approach in the treatment of vertebral metastases due to breast cancer. The aim of the study is to identify the prognostic factors associated with survival in breast cancer patients with spinal metastatic disease.

Methods

A retrospective cohort study was carried out at the National Cancerology Institute (INCAN) in Mexico City from January 2011 to December 2017. To this extent, 56 consecutive cases of patients with breast cancer were included. Multiple demographic, laboratory, and clinical variables were taken into account for the survival calculation. Kaplan-Meier graphs and log-rank tests were performed to observe significant differences by subgroups in survival, and Cox regression was used for multivariate analysis.

Results

Concerning the survival analysis, the patients who presented extra-spinal metastases, an unstable spine, and Frankel grade C had a statistically significantly worse prognosis. In the multivariate analysis, the variables included extra-spinal metastases, age >50 years, spinal instability, serum alkaline phosphatase, and CA 15.3 serum levels, finding statistical significance with a p=0.015.

Conclusion

Prognostic factors associated with shorter overall survival in breast cancer patients with metastatic spinal disease were the presence of extra-spinal metastases and spinal instability. Additionally, the use of the Tomita and Tokuhashi scores for patients with breast cancer and spinal metastases is not justified at present. The study should be continued with a larger population to decrease biases and obtain a more homogeneous sample, as well as to obtain a personalized score to determine a more efficient treatment for these patients.

## Introduction

The spine is the third most frequent site of metastasis, after the lungs and liver [1]. In studies decades ago, the frequency of vertebral metastases in autopsies of patients who died from cancer was estimated to be between 36% and 70% [2]. Due to the increased survival of patients with cancer as a result of a better treatment strategy for the primary tumor and early imaging diagnosis [3], the presence of vertebral metastases is now up to 70% and metastatic compression of the spinal cord is up to 10% in patients with vertebral metastases [4,5].

In the National Cancerology Institute (INCAN), a national reference institution in Mexico, the primary tumor most frequently associated with vertebral metastases is breast carcinoma, followed by prostate and lung cancer. The most common presentation in these cases is the multiple vertebrae presentation, with the thoracic and lumbar spines being the most affected segments. With the advance in systemic cancer therapy in general, it is expected that the number of patients with vertebral metastases will increase considerably, with metastatic spinal disease and metastatic compressive spinal cord injury being entities of great importance due to their associated high morbidity and mortality [6].

Nowadays, the choice of treatment for these patients is based on the prognosis, which is calculated considering multiple clinical features. Throughout time, several prognostic scoring systems have been proposed; the most commonly used are the Tomita score [7], the Tokuhashi score [8], and recently the neurologic, oncologic, mechanical, and systemic (NOMS) framework algorithm [9]. The treatment modality is decided based on those classifications, which can be surgery, radiotherapy, or both. Despite the multiple survival prognostic scores available, the therapeutic decision and the management of these patients are controversial; for this reason, it is important to individualize according to the patient and the resources of the institution.

## Materials and methods

Study design and patients population

It is also essential to note that these scoring systems are for vertebral metastases, regardless of any type of primary tumor, which seem not to be totally accurate at present due to the difference in survival between the various histological types and existing subtypes. The primary objective of this paper is the identification of clinical and epidemiological characteristics prevalent in patients with breast cancer and spinal metastases. The emphasis was placed on calculating survival rates and correlating them with epidemiological and clinical attributes. The derived results, focused on survival rates, were categorized based on the Tokuhashi and Tomita scores.

A retrospective cohort study was executed at the National Cancerology Institute, Mexico City, over a period spanning from January 2011 to December 2017. Included in this study were women aged 18 and above who were diagnosed with breast cancer accompanied by spinal metastases and had not undergone previous metastasis treatment at another medical facility. The exclusions encompassed patients with dual primary tumors or a history of spinal cord injuries, regardless of etiology.

Patients showing sequels of motor, sensory, or autonomic alteration due to degenerative, inflammatory, or infectious neurological diseases were also excluded. Additional exclusion criteria were the absence of follow-up, non-adherence to treatment, and the lack or inconclusiveness of histopathological reports.

Data collection and variables

Potential prognostic factors identified are age, presence and quantity of extraspinal metastases, incidence of Luminal Type and HER 2 status, specific location of spinal metastases, elevated serum levels of alkaline phosphatase and CA15-3, scores from assessments (such as Spinal Instability Neoplastic Score (SINS), Tokuhashi, Tomita, and Frankel), presence of pathological fractures, and history of radiotherapy. An organized spreadsheet, created using Microsoft Excel (Microsoft Corporation, Redmond, Washington, United States), was employed for meticulous data entry.

Statistical analysis

Descriptive statistics were utilized, represented as mean ± standard deviation or percentage as per relevance. Kaplan-Meier curves alongside the log-rank test facilitated the observation of significant survival disparities across subgroups. For a multivariate analysis, Cox regression was employed, focusing on risks and their associative influence on survival. The statistical analyses were conducted using IBM SPSS Statistics for Windows, Version 24 (released 2016; IBM Corp., Armonk, New York, United States), considering a p-value of 0.05 or less as the threshold for statistical significance.

## Results

A total of 56 consecutive cases of patients with breast cancer and vertebral metastases were included, of which 100% were female. The mean age was 53.64 ± 12.62 years, and 53.6% were >50 years old. Additionally, 91.1% had multiple vertebral lesions, 76.8% presented extra vertebral metastases at the time of diagnosis, 37.5% had spine instability according to the SINS score (score ≥ 8), and the Karnofsky score at the time of diagnosis was 76.6±14.43, with a score of >70 in 83.9% of the patients. Regarding the Frankel score, the percentages obtained were A: 1.8%, B: 3.6%, C: 7.1%, D: 10.7%, and E: 76.8%. Moreover, 42.9% of patients suffered pathological fractures. The most frequent type of bone lesion was blastic in 37.5%, followed by lytic in 35.7%, and mixed in 26.8%.

**Figure 1:**
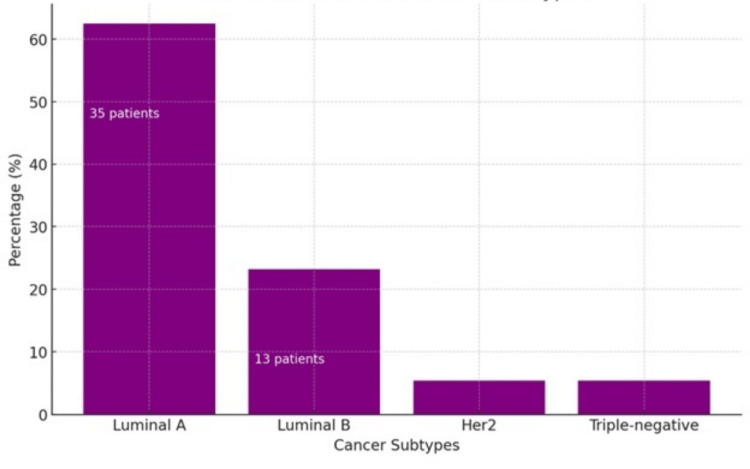
Distribution of breast cancer subtypes in the study population

Concerning treatment, 75% of the patients had previously received radiotherapy for vertebral lesions; the most affected vertebral segment was the thoracic with 85.7%, followed by the lumbar with 75%, the cervical with 28.6%, and the sacrum with 28.6%. Furthermore, the mean serum levels of CA 15-3 and alkaline phosphatase were 386.56 ± 406 IU/mL and 179.94 ± 177 IU/L, respectively. For the previously mentioned serum level values, cut-off points of 35 IU/mL and 126 IU/L were taken for subsequent subgroup analysis, respectively. As for the different phenotypes, it was observed that from all the patients, the luminal A phenotype corresponded to the majority with 62.5% (35 patients), luminal B to 23.2% (13 patients), human epidermal growth factor receptor 2 (HER2) to 5.4% (three patients), and triple-negative to 5.4% (three patients) (Figure 1).

**Figure 2:**
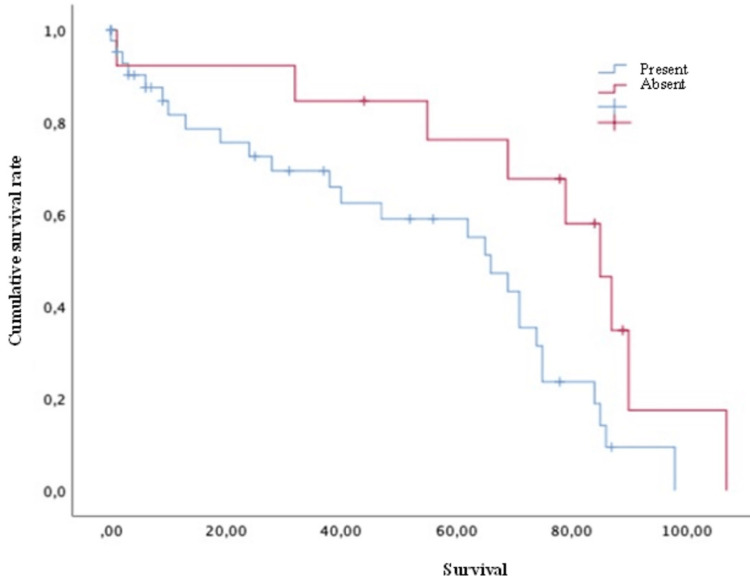
Survival of breast cancer patients with spinal metastases and with or without extra vertebral metastases Kaplan-Meier Curve. Statistically significant differences were observed between subgroups. Results obtained were: present=53.76; absent=74.92 (p=0.027)

**Figure 3:**
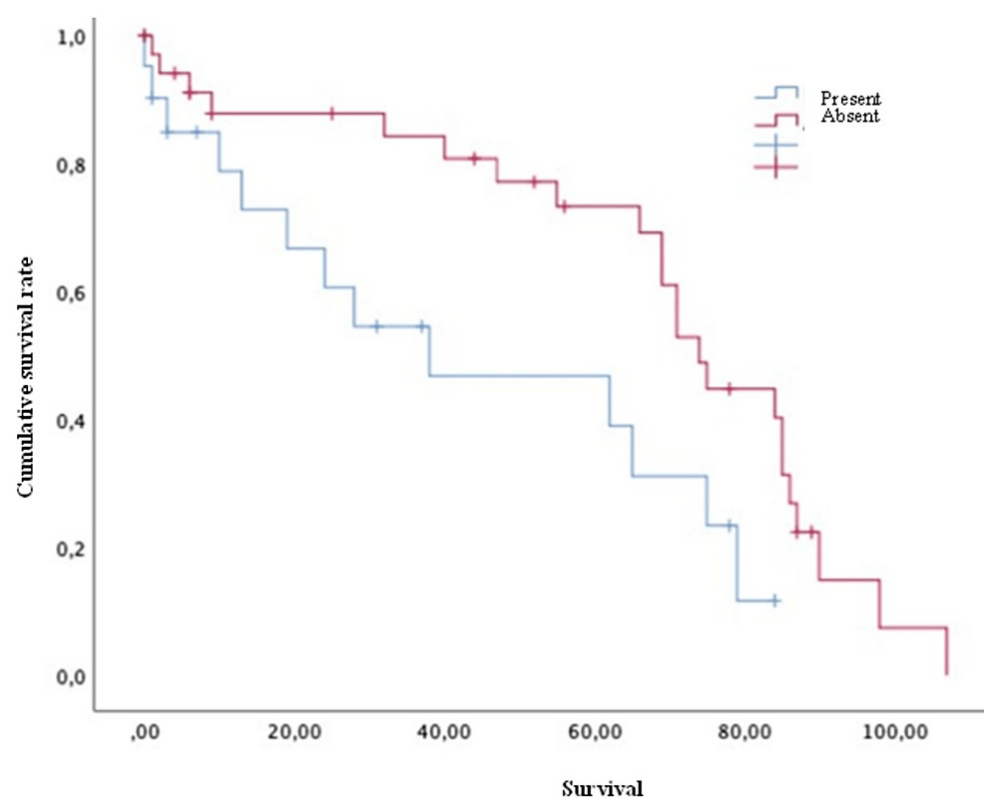
Survival of breast cancer patients with spinal metastases and with or without spinal instability Kaplan-Meier Curve. Statistically significant differences were observed between subgroups. Results obtained were: present=43.69; absent=68.22 (p=0.019)

Concerning survival, the luminal A group showed a mean of 61.79 months, luminal B of 66.08 months, HER2 of 75 months, and triple-negative of 39.3 months. Finally, concerning the survival analysis, the patients with extraspinal metastases (Figure 2), unstable spines (Figure 3), and Frankel grade C (statistically significant differences with results demonstrating: A=62; B=54.5; C=16.75; D=81; and E=61 [p = 0.030]) had a statistically significantly worse prognosis. The mean survival of the patients, in general, was 59.90 ± 4.89 months.

**Figure 4:**
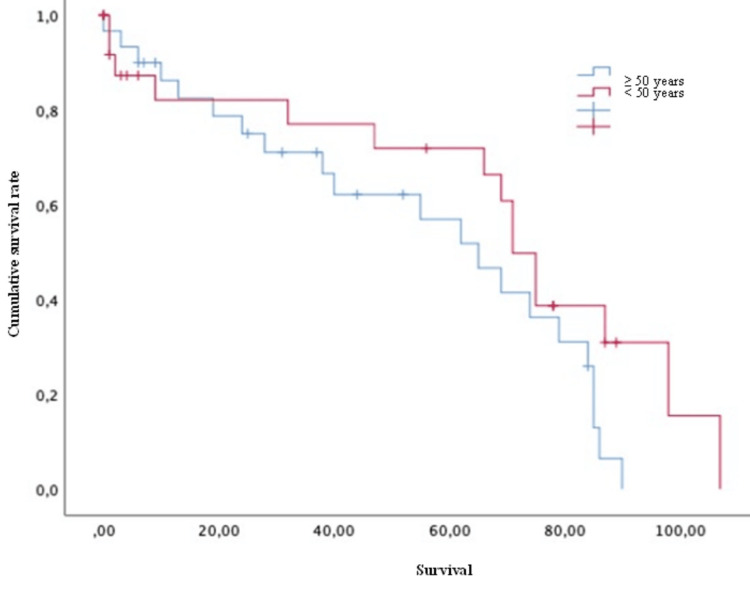
Survival of patients with breast cancer and spinal metastases according to age (years) Kaplan-Meier Curve. No statistically significant differences were observed between subgroups. Results obtained were: present=55.03; absent=66.78 (p=0.112)

**Figure 5:**
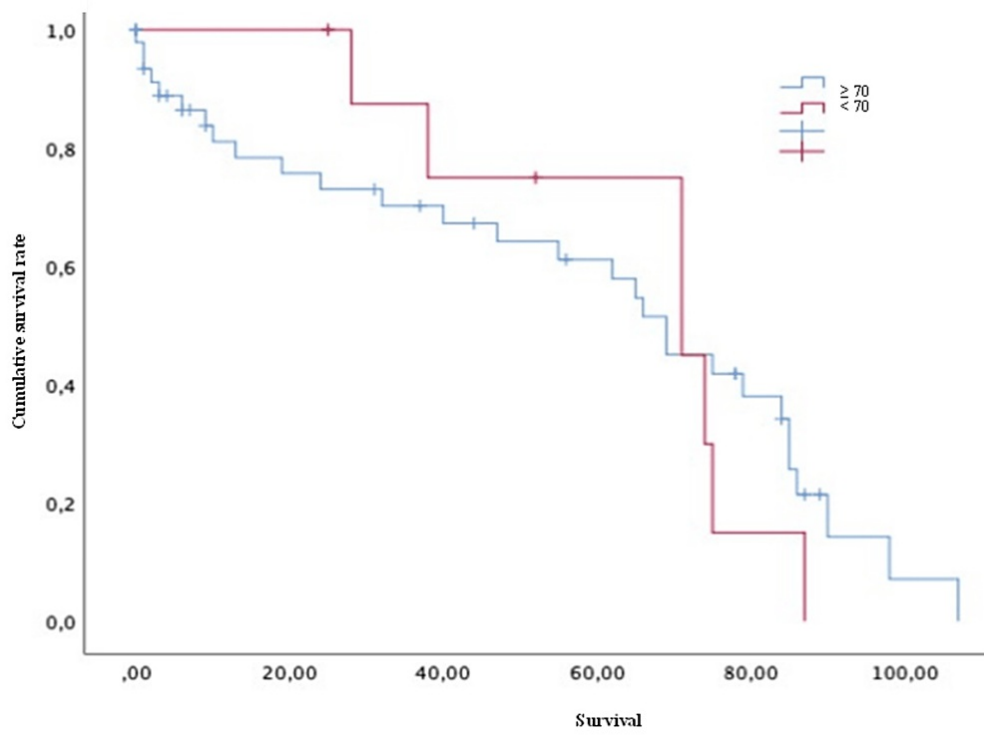
Survival of patients with breast cancer and spinal metastases according to Karnofsky score Kaplan-Meier Curve. No statistically significant differences were observed between subgroups. Results obtained were: present=58.79; absent=64.95 (p=0.742)

**Figure 6:**
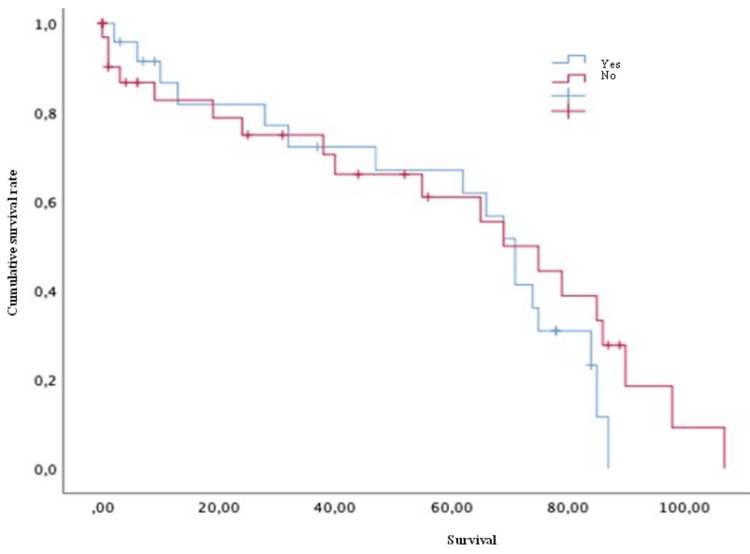
Survival of patients with breast cancer and spinal metastases with pathological fractures Kaplan-Meier Curve. No statistically significant differences were observed between subgroups. Results obtained were: present=58.4; absent=61.17 (p=0.361)

**Figure 7:**
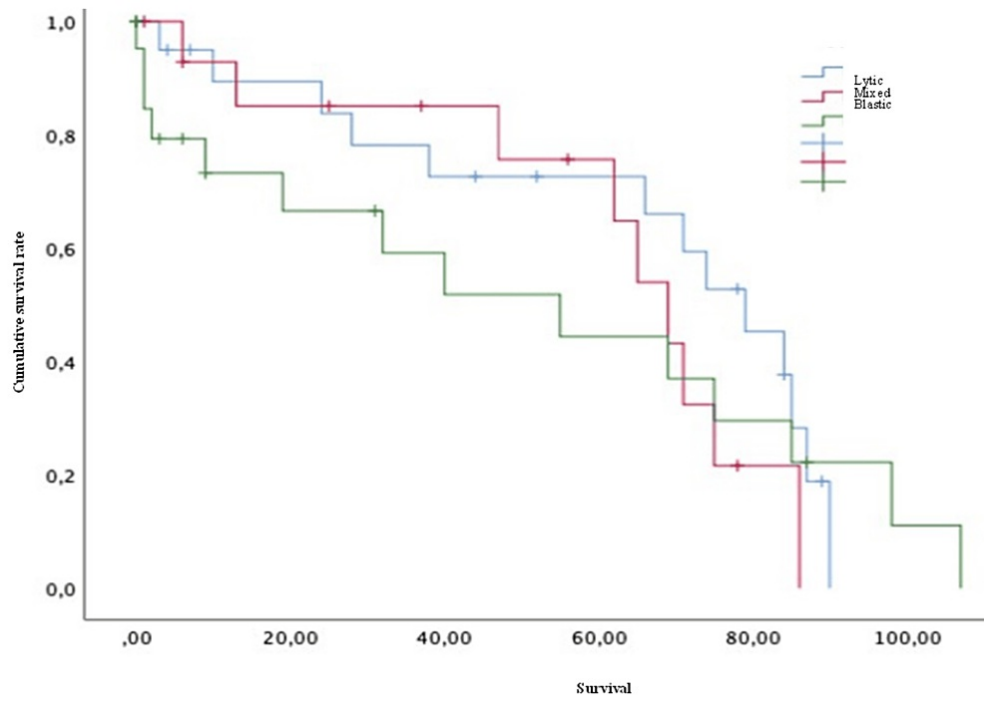
Survival of patients with breast cancer and spinal metastases by type of bone lesion Kaplan-Meier Curve. No statistically significant differences were observed between subgroups. Results obtained were: lytic=65.18; mixed=61.43; blastic=51.12 (p=0.726)

**Figure 8:**
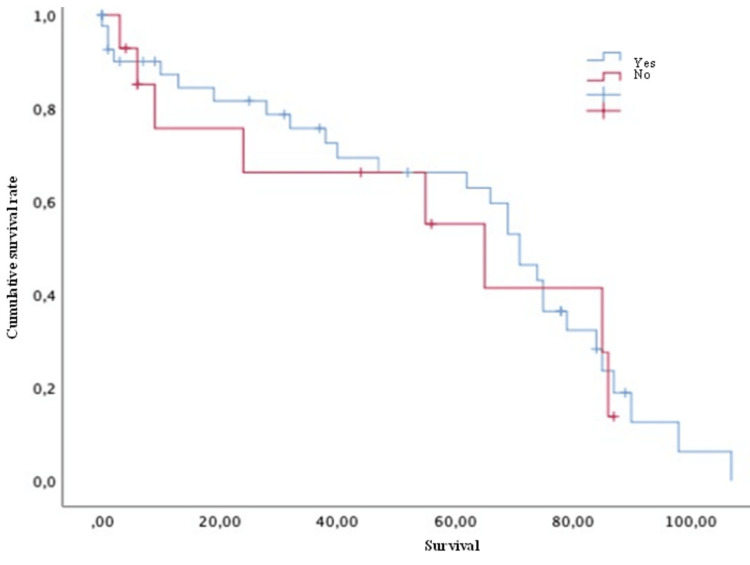
Survival of patients with breast cancer and spinal metastases with previous radiotherapy Kaplan-Meier Curve. No statistically significant differences were observed between subgroups. Results obtained were: yes=61.20; no=54.41 (p=0.745)

**Figure 9:**
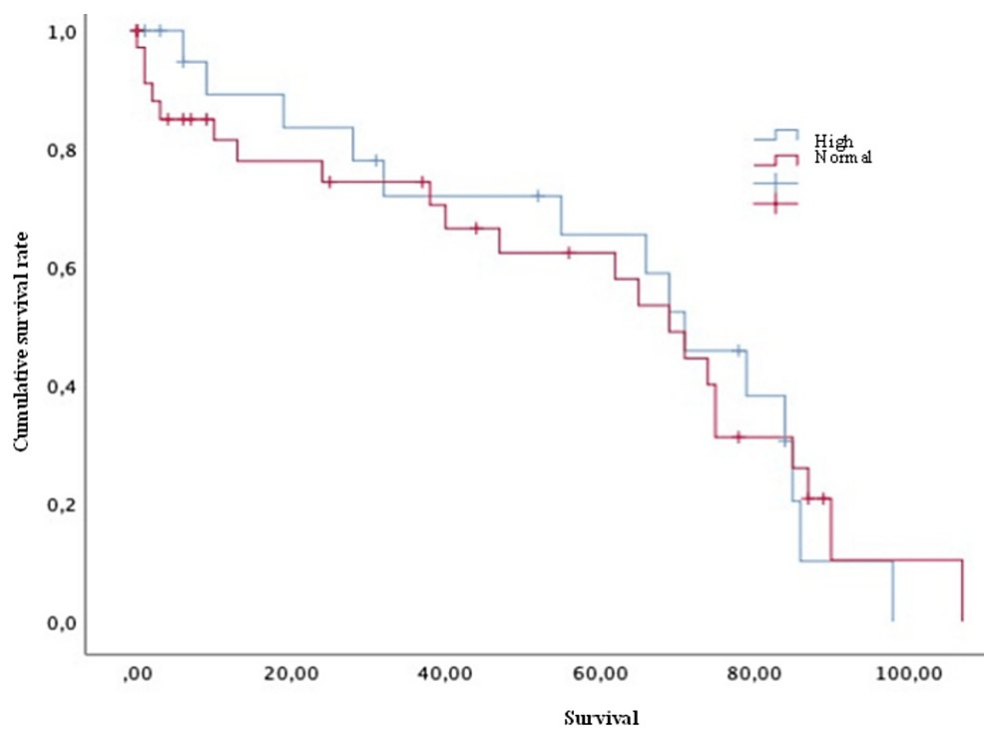
Survival of patients with breast cancer and spinal metastases, and alkaline phosphatase levels Kaplan-Meier Curve. No statistically significant differences were observed between subgroups. Results obtained were: yes=62.29; no=58.191 (p=0.975)

**Figure 10:**
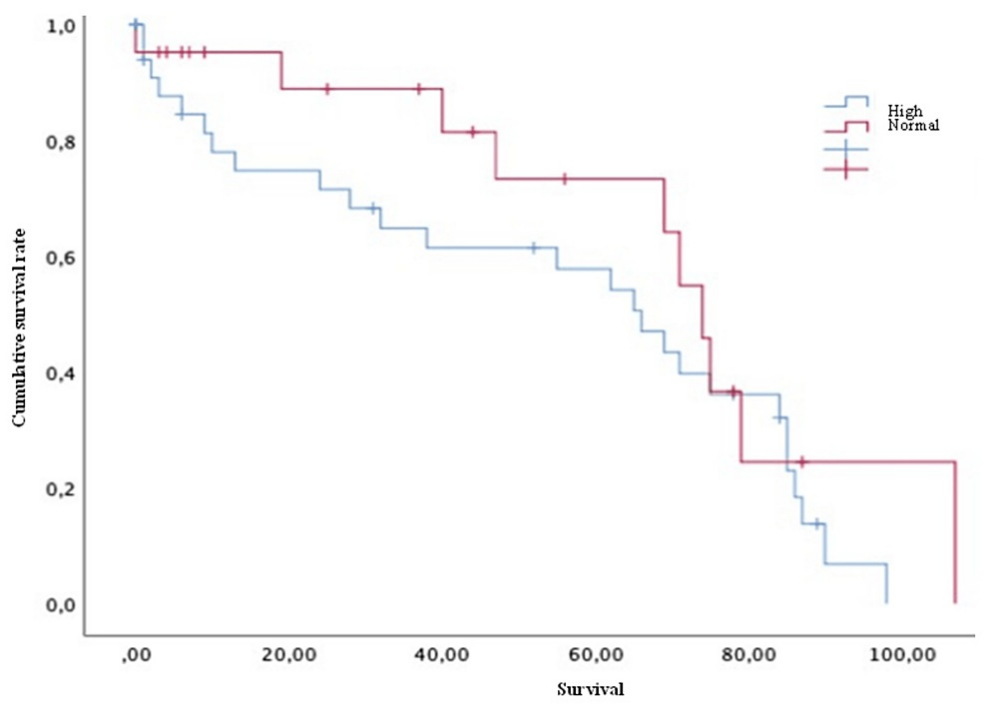
Survival of patients with breast cancer and spinal metastases, and CA 15.3 levels Kaplan-Meier Curve. No statistically significant differences were observed between subgroups. Results obtained were: yes=54.33; no=70.30 (p=0.229)

**Figure 11:**
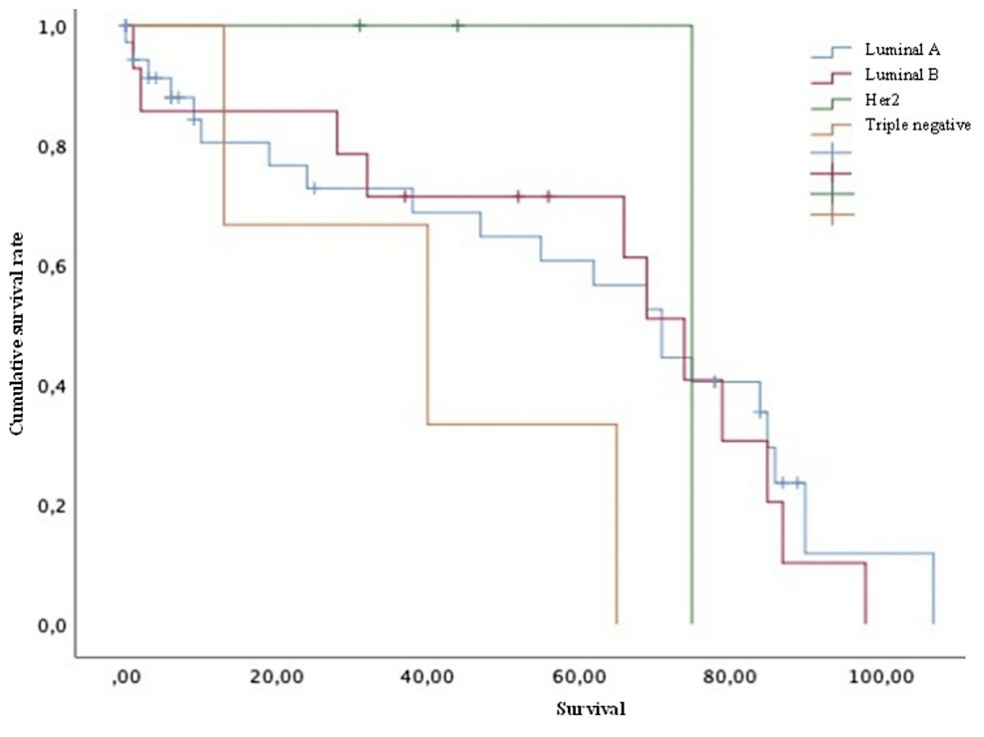
Survival of patients with breast cancer and spinal metastases according to phenotype Kaplan-Meier Curve. No statistically significant differences were observed between subgroups. Results obtained were: luminal A=59.99; luminal B=61.43; HER2=75.00; triple negative=39.33 (p=0.280) HER2: human epidermal growth factor receptor 2

Conversely, variables such as age (both > or < 50 years old) (Figure 4), Karnofsky score (both > or <70) (Figure 5), the presence of pathological fractures (Figure 6), the type of lesion (lytic, blastic, or mixed) (Figure 7), previous radiotherapy (Figure 8), the location of the spinal lesion (cervical, thoracic, lumbar, or sacral), elevated values of alkaline phosphatase (Figure 9), elevated values of CA 15-3 (Figure 10), and the different phenotypes (Figure 11), were not associated with a worse prognosis in a statistically significant way.

**Figure 12:**
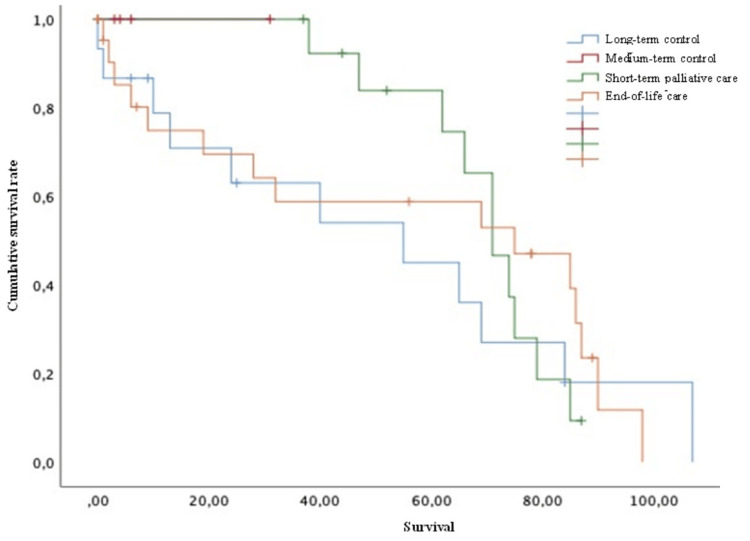
Survival of patients with breast cancer and spinal metastases according to Tomita score Kaplan-Meier Curve. No statistically significant differences were observed between subgroups. Results obtained were: long-term local control=39.46; medium-term local control=11.00; short-term palliation=59.20; terminal care=45.40 (p=0.745)

According to the Tomita score, the patients were distributed as follows: 1) long-term control (50 months survival) corresponded to 26.8% of the patients; 2) local control in the medium term (23.5 months survival) to 7.1%; 3) short-term palliative (15 months survival) to 26.8%; and 4) terminal care (six months survival) to 39.3% (Figure 12).

**Figure 13:**
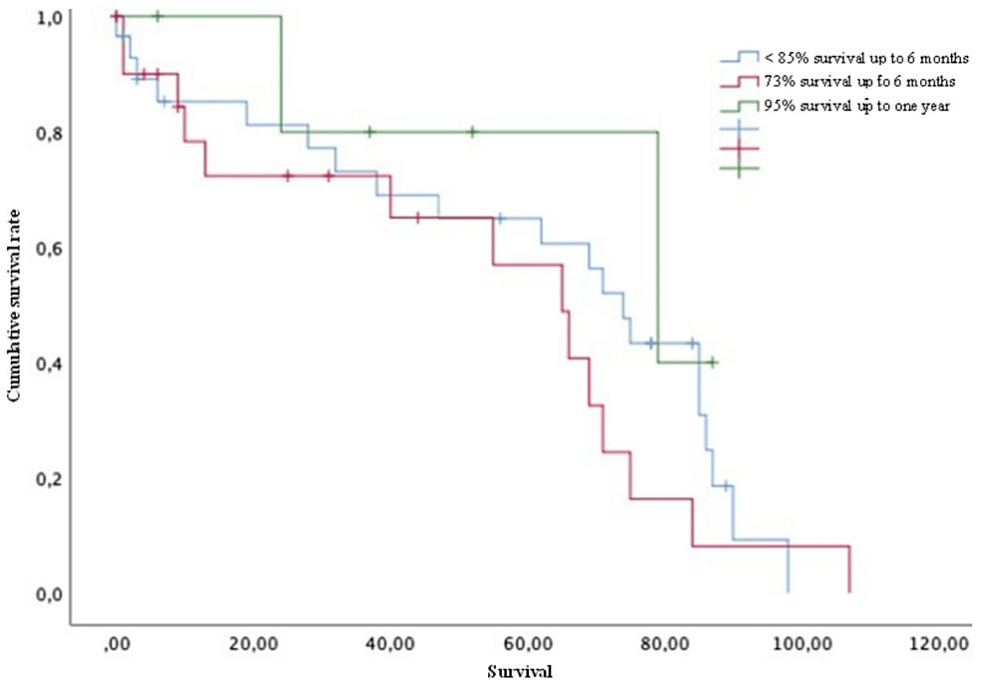
Survival of patients with breast cancer and spinal metastases according to Tokuhashi score Kaplan-Meier Curve. No statistically significant differences were observed between subgroups. Results obtained were: <6 months survival=60.93; >6 months survival=53.05; >1 year survival=71.20 (p=0.359)

In the same way, according to the Tokuhashi score, patients were distributed as follows: 1) survival of <85% up to six months corresponded to 51.8% of the patients; 2) survival of 73% up to six months corresponded to 37.5%; 3) survival of 95% up to one year corresponded to 10.7% of all patients. No significant statistical differences were found between the groups (Figure 13).

After univariate analysis, extraspinal metastases, >50 years of age, spinal instability, elevated serum alkaline phosphatase, and elevated serum CA 15.3 were included in the multivariate analysis (Table 1).

**Table 1:** Cox logistic regression for multivariate analysis

Variable	Hazard ratio	p Value
Extraspinal metastases	2.756	0.016
Age 50 years	1.601	0.216
Spinal instability	1.483	0.050
Alkaline phosphatase value	0.909	0.609
CA 15-3 value	1.156	0.156

## Discussion

Patients with breast cancer and spinal metastases were included in this study. The epidemiological variables measured behaved in a very similar way to those reported in the world literature [10]. The segment that was most frequently affected was the thoracic, probably due to its more significant proportion. However, other factors could be involved. Still, there are no reports in the literature to justify this association for any other reason. It is essential to highlight that, at the time of spinal metastasis diagnosis, 37.5% of the patients presented with spinal instability. In the latter cases, adequate patient selection for surgery treatment is essential since, once spinal instability is considered, mortality increases significantly, as observed in the presented survival analysis. Furthermore, neurological functional status measured by the Frankel scale was associated with a statistically significant shorter survival when classified as C. Despite that, since the distribution was not homogeneous, these results must be taken cautiously. Further studies will be needed to address this possible correlation.

Moreover, the types of metastatic bone lesions were not related to differences in survival, despite previous studies that described an association. Additionally, as previous results reported in the world literature indicate, the patient´s survival was around 60 months [10,11]. Furthermore, although there was a tendency for patients with higher CA 15.3 values to have shorter overall survival, this was not statistically significant and probably depended on vertebral tumor burden in order to have higher expression. On the other side, alkaline phosphatase values did not have any relation to survival. The cut-off values were the parameters used by the National Cancerology Institute; however, it is reason for another study to standardize cut-off values that may be related to survival in this specific type of patient.

In the survival analysis by phenotypes, a clear trend of shorter overall survival can be observed in patients with triple-negative phenotypes. Nonetheless, no significance is given due to the limited number of patients in this sample group. On the other hand, the variables with statistical significance for a shorter overall survival were the presence of extra-spinal metastases and spine instability; therefore, these variables were taken to perform a multivariate analysis. In the latter, statistically significant less survival was observed when the patients jointly presented extra-spinal metastases (2.7 times more risk), age greater than 50 years, spinal instability, and elevated CA 15.3 values for extraspinal metastasis and spinal instability, those that are more significantly related to shorter overall survival.

**Figure 14:**
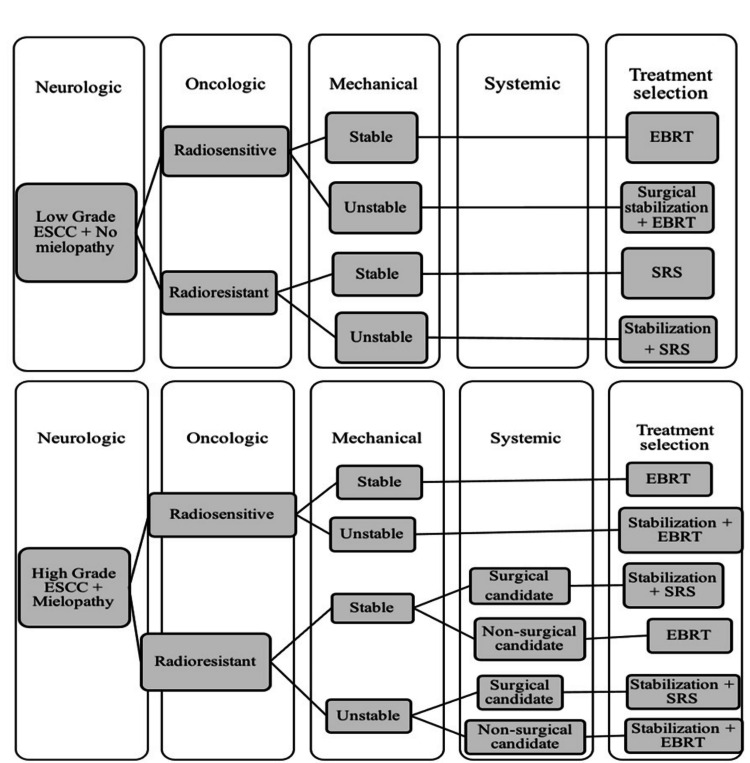
NOMS Management algorithm for vertebral metastases Here we report and provide the evidence for the neurologic, oncologic, mechanical, and systemic (NOMS) decision framework

The most common prognostic scoring systems are the Tomita score [7], the Tokuhashi score [8], and recently the NOMS algorithm [9] (Figure 14).

It was also noted that when our patients were analyzed in terms of the Tomita and Tokuhashi scores, there was no tendency to behave according to what is described in those scores. On top of that, no statistically significant differences between the subgroups were observed, so using these scores is not currently justified for these patients.

Variables associated with survival in patients with breast cancer and vertebral metastases

As mentioned above, vertebral metastases due to breast cancer are the most frequent type of metastases. There is a broad difference in the survival of these patients compared to other neoplasms that generate spinal metastases [9]. Therefore, there must be a specific score for breast cancer patients, taking into consideration their peculiarities and differences.

Many attempts have been made to associate different types of variables with the prognosis of patients with vertebral metastases from breast cancer. Chen et al. proposed important risk factors associated with the probability of developing bone metastases in this group of patients, finding that those who presented lymph node metastases, elevated serum levels of CA 15.3, CA 125, and alkaline phosphatase had a greater risk of presenting bone metastases in a statistically significant manner [10]. However, patients with metastatic bone disease were taken into account regardless of the bone site. It is therefore unknown if it follows the same behavior if only vertebral metastases are considered. Moreover, the presence of growth factor receptors has been an important area of interest for the study and treatment of these patients. Lin et al. [11] found that the presence of progesterone receptors was related to the presence of blast vertebral lesions and a worse prognosis. Also, Wang M. et al. analyzed the relationship between the survival of these patients with different phenotypes. Negative and triple-negative estrogen receptors had a statistically significant shorter overall survival, being even shorter in the latter group [12]. Furthermore, the biomechanical spine features could also be related to the survival of this group of patients. In a study by Mostafa et al., the relationship between survival and the measurement of the psoas muscle diameter was calculated in these patients. It was found that the mean survival was 104 days and that patients with a smaller diameter of the psoas muscle presented a statistically significant shorter survival, proposing this finding as a poor but new possible prognostic factor [13].

On the other hand, Schlamp et al. [14] discovered a relationship between the survival of these patients and their spinal stability after radiotherapy treatment, finding that patients presented improvement in stability six months after radiotherapy. However, there were no statistically significant differences in terms of stability and survival. Foerster et al. [15] proposed some other survival prognostic factors in patients with breast cancer and vertebral metastases with spinal instability. They found that patients older than 50 years with multiple spinal metastases and the triple-negative subtype presented a statistically significant worse prognosis. The vertebral portion involved and the extent of metastasis could also influence the prognosis of the patients.

Additionally, Guzik et al. [16] analyzed the different morphologies of vertebral metastases according to the Tomita vertebral tumor morphology scale. They noticed that the location and involvement of spinal metastases are related to a lower quality of life: the greater the portion of the vertebra involved, the lower the quality of life of the patient. Notably, none of these studies takes into account the SINS scale, which is currently the standard for classifying whether the tumor-bearing spine is stable or unstable. On the other hand, the revised Tokuhashi score and its adaptation to be individually used in patients with vertebral metastases and breast cancer have been previously evaluated. A mean survival of 24 months was found. The triple-negative type was associated with shorter survival, as well as negative estrogen and progesterone receptors and the HER 2 phenotype, although to a lesser extent, the last two were mentioned. It was also found that the Tokuhashi score without the aforementioned adaptation is not related to the prognosis of this kind of patient [17].

In our study, we observed that the Tomita and Tokuhashi scores do not have a relationship with the general survival of our patients (regardless of the primary tumor), as they are neither updated in terms of the variables nor tumor-specific. It is therefore probably not related to the survival of our patients with breast cancer and vertebral metastases.

Surgical intervention in spinal metastases

Central to our observations was the pivotal role of surgical considerations, especially in cases marked by spinal instability, where mortality rates were notably amplified. Modern surgical advancements, such as the incorporation of microscopic and exoscopic techniques, have heralded nuanced precision and visibility, optimizing the surgical outcomes for metastatic spine lesions [18,19]. These technological adaptations facilitate enhanced meticulousness in surgical procedures, minimizing collateral damage to surrounding tissues and optimizing tumor resection, which is pivotal for mitigating subsequent complications and enhancing post-surgical recuperation trajectories. However, the essence of surgical success remarkably hinges on strategic patient selection, underscoring the indispensable nature of comprehensive pre-surgical evaluations. This includes a scrupulous assessment of spinal stability and the potential influence of surgical interventions on overall survival and quality of life.

Surgical and radiotherapeutic intersection

One of the compelling revelations was the fundamental role of surgical strategies, especially in cases marked by pronounced spinal instability. Concurrently, radiotherapy has emerged as a crucial therapeutic ally in the management arsenal. Modern advancements in radiotherapeutic approaches, specifically precision-targeted modalities, have increasingly shown promise in optimizing the management of spinal metastases. Radiotherapy offers the potential benefit of managing symptomatic vertebral metastases, improving pain control, and enhancing structural integrity.

In the intertwined landscape of surgery and radiotherapy, strategic integration appears vital. The meticulous employment of microscopic and exoscopic surgical technologies enhances the precision of tumor resection and mitigates collateral tissue damage. Following surgical intervention, radiotherapy acts as a complementary force, aimed at obliterating residual microscopic disease, mitigating the risk of local recurrence, and bolstering overall therapeutic efficacy. The integration of technological foresight such as microscopic and exoscopic visualization within surgical paradigms represents a transformative evolution. When paralleled with advanced radiotherapeutic strategies, a comprehensive and multidimensional therapeutic approach is cultivated, enhancing the specificity and effectiveness of treatment protocols [20,21].

Neurological implications, phenotypic variations and survival outcomes

The study unveiled nuanced correlations between neurological status, primarily gauged through the Frankel scale, and survival rates. It evinced that a classification of ‘C’ on the Frankel scale was intricately intertwined with a diminished survival trajectory. The heterogeneous distribution within these classifications necessitates a judicious interpretation of the findings, underscoring the need for bolstered research endeavors for enriched insights.

The study further embarked on a meticulous exploration of survival trajectories across varied phenotypic manifestations. It unveiled a discernible trend of reduced survival in patients characterized by a triple-negative phenotype, although hampered by statistical insignificance owing to the limited sample pool.

Critical variables influencing survival

A synergistic analysis of multivariable influences revealed a constellation of variables, including extraspinal metastases and spinal instability, as being paramount influencers of survival trajectories. The technological foresight offered by microscopic and exoscopic methodologies emerged as transformative in navigating these complex surgical landscapes, marking a significant evolution in managing spinal metastases.

Limitations of the study

The main limitation of the study was the small sample size that could be expanded to reach a higher significance level, as well as the fact that it was a monocenter study. Also, the study didn’t include molecular studies to distinguish BRCA 1-2 expression, which could be a potential determinant factor in breast cancer prognosis. Including this data could provide a more comprehensive understanding of disease prognosis.

## Conclusions

Prognostic factors associated with shorter overall survival in breast cancer patients with vertebral metastases were found to be the presence of extra-spinal metastases and spinal instability. The presented results are of medical importance as these variables are not being considered by any of the scores currently available. Further acknowledgement and use of these variables could allow a more realistic categorization and better treatment for this group of patients. Thus, the use of both the Tomita and Tokuhashi scores for patients with breast cancer and spinal metastases seems not justified at present. The study of the currently found prognostic variables for patients with breast cancer and spinal metastases should be continued with a larger population in order to correct biases and obtain a more homogeneous sample.
